# Health versus other sectors: Multisectoral resource allocation preferences in Mukono district, Uganda

**DOI:** 10.1371/journal.pone.0235250

**Published:** 2020-07-30

**Authors:** Tatenda T. Yemeke, Elizabeth E. Kiracho, Aloysius Mutebi, Rebecca R. Apolot, Anthony Ssebagereka, Daniel R. Evans, Sachiko Ozawa

**Affiliations:** 1 Division of Practice Advancement and Clinical Education, UNC Eshelman School of Pharmacy, University of North Carolina, Chapel Hill, NC, United States of America; 2 Department of Health Policy Planning and Management, Makerere University School of Public Health, Makerere University, Kampala, Uganda; 3 Duke University School of Medicine, Durham, NC, United States of America; 4 Department of Maternal and Child Health, UNC Gillings School of Global Public Health, University of North Carolina, Chapel Hill, NC, United States of America; University of Vermont, UNITED STATES

## Abstract

**Objectives:**

To elicit citizen preferences for national budget resource allocation in Uganda, examine respondents’ preferences for health vis-à-vis other sectors, and compare these preferences with actual government budget allocations.

**Methods:**

We surveyed 432 households in urban and rural areas of Mukono district in central Uganda.We elicited citizens’ preferences for resource allocation across all sectors using a best-worst scaling (BWS) survey. The BWS survey consisted of 16 sectors corresponding to the Uganda national budget line items. Respondents chose, from a subset of four sectors across 16 choice tasks, which sectors they thought were most and least important to allocate resources to. We utilized the relative best-minus-worst score method and a conditional logistic regression to obtain ranked preferences for resource allocation across sectors. We then compared the respondents’ preferences with actual government budget allocations.

**Results:**

The health sector was the top ranked sector where 82% of respondents selected health as the most important sector for the government to fund, but it was ranked sixth in national budget allocation, encompassing 6.4% of the total budget. Beyond health, water and environment, agriculture, and social development sectors were largely underfunded compared to respondents’ preferences. Works and transport, education, security, and justice, law and order received a larger share of the national budget compared to respondents’ preferences.

**Conclusions:**

Among respondents from Mukono district in Uganda, we found that citizens’ preferences for resource allocation across sectors, including for the health sector, were fundamentally misaligned with current government budget allocations. Evidence of respondents’ strong preferences for allocating resources to the health sector could help stakeholders make the case for increased health sector allocations. Greater investment in health is not only essential to satisfy citizens’ needs and preferences, but also to meet the government’s health goals to improve health, strengthen health systems, and achieve universal health coverage.

## Introduction

Low health sector spending and investment is a perennial challenge, especially in low and middle-income countries (LMICs) [1]. Many LMICs consistently allocate resources to the health sector at levels that are well below what is considered optimal by various multilateral conventions [2]. For example, despite governments of Africa committing to allocate at least 15% of their budgets toward the health sector in the Abuja declaration, public financing of the health sector remains much lower in Africa [3, 4]. External donor funding and development assistance has long supplemented the health financing gaps caused by low domestic spending. However, growth in external funding for health has recently slowed [5], and there is need for governments to mobilize more domestic resources for health. Inadequate health sector spending creates weak health systems with poor health outcomes, threatens the attainment of universal health coverage [6], and thwarts progress towards the United Nations’ Sustainable Development Goals to ensure good health and wellbeing [7]. Furthermore, inadequate investment in health has been linked with slower economic growth and lower levels of human capital [8].

Efforts to create more fiscal space for the health sector occur in the context of competing demands from other sectors and political contest [9]. Hence, there is need to generate evidence to support increased resource allocation to the health sector. Understanding citizens’ preferences for resource allocation and valuation of health can be a powerful tool for policy makers and other stakeholders to meet voters’ demands, make evidence-based decisions, and collectively set priorities [10–13]. Evidence of citizens’ preferences for resource allocation for health can be used to make the case for increased allocation to the health sector and incorporate their preferences in decision-making to improve overall social welfare [14]. However, there is little such evidence available in LMICs [15].

Best-worst scaling (BWS) is an elicitation method that has been used to measure people’s preferences, including health related choices and policy problems requiring prioritization [16–20]. For example, BWS has been utilized to measure patient and caregiver health preferences to guide clinical decision making [21–23], inform health interventions [24, 25], and measure public preferences for funding health technologies [19]. Instead of ranking their preferences for the full choice set at once, BWS survey respondents select their most and least preferred choices in a series of smaller choice tasks, which are a subset of all the choices [17]. BWS survey responses are then analyzed to obtain a ranked list of preferences for the whole choice set [17]. BWS surveys have the advantage of being less cognitively challenging for respondents, thus easier to administer compared to other discrete choice experiments, and their results can be comprehended by a broader range of stakeholders [26, 27]. BWS is thus suited for eliciting preferences across the general population with varying levels of literacy and socio-economic status in LMIC contexts. This study adds to the few published studies eliciting citizens’ preferences for resource allocation in the African region [28].

In Uganda, a low-income country, health sector spending at 7.3% of the gross domestic product (GDP) is considered below the optimal level needed to meet the needs of the health system [29]. Notwithstanding potential improvement to fiscal space for the health sector from greater efficiency and reduction in wasted resources, a World Bank report concluded that Uganda needs to allocate more resources to the health sector to meet its health goals [30]. This study elicits Ugandan citizens’ preferences for resource allocation across all sectors using a BWS survey, and examines how their stated preferences compare with government budget allocations.

## Methods

### Study setting and sampling

We conducted a cross-sectional survey in Uganda to elicit respondents’ preferences for national budget resource allocation, comparing preferences for health to other sectors. The study was conducted in Mukono district in central Uganda in December 2017. To examine differences between rural and urban areas, we used simple random sampling to select one of two urban divisions (Mukono Central division) and one rural sub-county (Seeta Namuganga). In the rural area, two villages (Nsagi and Mawotto) were randomly selected from two parishes. In the urban area, one zone was randomly selected from each of the two wards in the municipality: Agip zone from Gulu ward and Upper Kauga zone from Nsubbe Kauga ward. A comprehensive listing of households in each village/zone was obtained with the help of Village Health Team (VHT) members and community leaders. Our sample size was allocated across the four selected study areas based on probability proportional to size (i.e. Agip– 109 households, Upper Kauga– 108 households, Mawotto– 98 households, and Nsagi 117 households). One member of a household, either the head of household or their spouse above 18 years of age, was eligible to participate in the study.

### Study instrument

The study instrument consisted of a BWS survey to elicit respondents’ preferences for resource allocation across government sectors and questions on respondents’ demographic and other background characteristics (see S1 File). Sixteen sectors, corresponding to the Uganda national budget line items, were included in the BWS survey: (1) health; (2) water and environment; (3) education; (4) agriculture; (5) works and transport; (6) social development; (7) security; (8) energy and mineral development; (9) public sector management; (10) accountability; (11) justice, law and order; (12) information and communication technology; (13) lands, housing and urban development; (14) tourism, trade and industry; (15) public administration; and (16) legislature.

**Fig 1:**
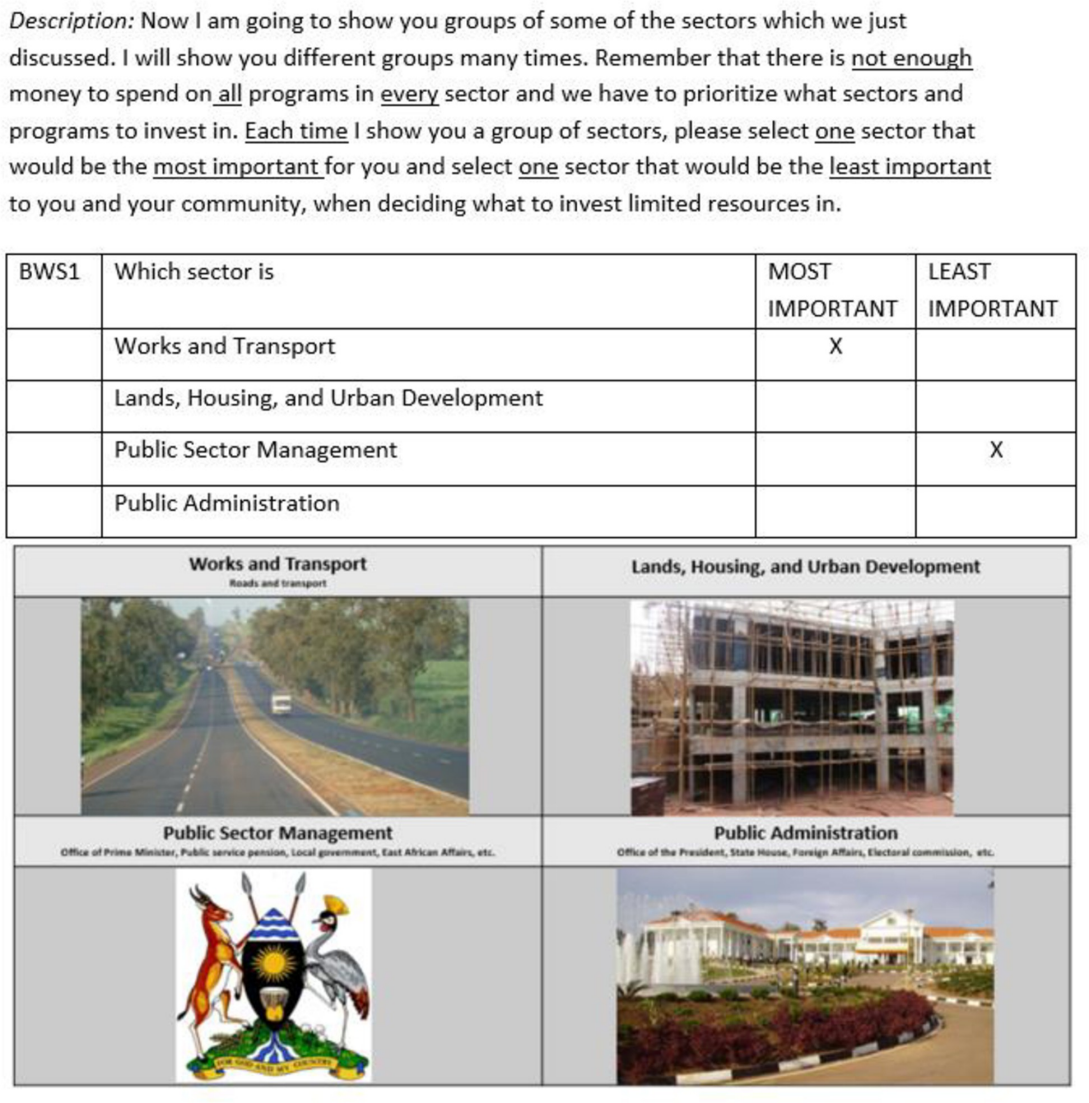
Example best worst scaling choice task and visual aid.

Using the MaxDiff package in R software [31] and a main effects orthogonal design [32], we generated 16 choice tasks, where each choice task contained four sectors, corresponding to the Uganda national budget line items (Fig 1). The orthogonal design implies that each sector appeared an equal number of times across choice tasks, and thus had an equal chance of being selected as the most or least important sector, where preferences for each sector could be measured independently [33]. For each of the 16 choice tasks, respondents selected the most and least important sector to allocate government resources to, from a list of four sectors in each choice task. To ensure that respondents understood each sector, we read out a detailed description of programs and functions of each sector. Further, to make it easier for respondents with lower literacy to understand the choice tasks, we developed accompanying visual aids, with pictures representing different sectors and accompanying text which were read out. We also included a warm-up choice task based on food preferences, to ease understanding of the tasks. All study materials were translated into Luganda (the local language spoken in Mukono district) and back-translated into English to check for accuracy. We conducted a pre-test of the study instrument in a village in Seeta Namuganga to assess respondents’ understanding of the BWS questions and tested the visual aids. The final instrument was modified where wording was tweaked to improve understanding based on feedback received from the pre-test.

### Data collection

Six trained Ugandan research assistants who conducted the pre-test administered the survey tools. VHTs and community leaders acted as guides, and identified the households that had been randomly drawn from the sampling list. All respondents provided written consent before participating in the study. Study data were collected and managed using REDCap (Research Electronic Data Capture) electronic data tools hosted at the University of North Carolina at Chapel Hill [34]. Respondents’ responses were recorded on electronic tablets using REDCap’s mobile application for offline data collection [35]. Data were synchronized to the REDCap server at the end of each data collection day after checking for completeness and accuracy. Ethical approval of the study was sought and obtained from the ethical review boards at the University of North Carolina at Chapel Hill, Makerere University School of Public Health, and the Uganda National Council for Science and Technology.

### Data analysis

We used Chi-squared and *t-*tests to examine whether there were any differences between urban and rural respondents’ demographic characteristics. The BWS data were analyzed using the relative best-minus-worst score method [21–23, 36, 37] and McFadden’s conditional logistic regression [38]. The relative best-minus-worst score method or count analysis produces similar results with more complex regression-based methods, but is easier to interpret for a broader range of stakeholders [37, 39, 40]. We counted the number of times each sector was chosen as the most important sector and the number of times it was chosen as the least important sector across all respondents. We then calculated a mean best-worst score for each sector by dividing the difference in the number of times a sector was chosen best minus worst, by the number of times a sector could have been selected (i.e. 4 times per respondent) across all respondents. The best-worst score represents a score between -1 and 1. A higher, positive mean best-worst score reflects that a sector was more frequently selected as the best sector to invest resources in and less frequently selected as the worst, and is therefore preferred relative to other sectors with lower mean best-worst scores. We calculated the standard errors of the mean best-worst scores and performed t-tests to assess whether the mean best-worst scores for each sector were significantly different from zero. We also examined if any observable differences in preferences existed between rural and urban populations. We then compared respondents’ preferences for resource allocation across sectors with actual government budget allocations. Specifically, the percentages of planned government expenditures in the 2017–2018 Uganda national budget fiscal year allocated to each sector were compared to the ranked preferences from our BWS survey. We transformed the mean best-worst scores into a positive scale anchored at zero and estimated the cumulative sums. Each sector in the BWS survey was allocated a percentage preference relative to these cumulative sums (see S1 Data).

For the regression analysis, we assumed sequential best-worst responses, i.e. respondents first chose the most important sector, and then chose the least important sector from the remaining sectors. Thus, the choice of the most important sector is independent of the choice of the worst sector, and the choice of the worst sector was conditioned on the choice of the best sector. The choice of the most important and least important sector was described via a single dichotomous dependent variable for each respondent and choice task. Using McFadden’s conditional logistic regression with effects coding [41, 42], we regressed the choice variable on all sectors. We estimated the coefficient of the omitted sector in effects coding as the negative sum of the non-omitted coefficients, and the standard error as the square root of the sum of the variance-covariance matrix from the initial regression [42].

## Results

A total of 432 respondents completed the BWS survey, including 217 in urban and 215 in rural areas of Mukono district. Table 1 summarizes the demographic and socio-economic characteristics of the respondents including *p* values of Chi-squared and *t-*tests comparing characteristics of urban and rural respondents. Rural respondents were less educated, represented more minority ethnic groups, were more Christian, and were of lower socioeconomic status than urban respondents. Our urban sample of respondents was younger, single, with more females than the rural sample. There were no significant differences in employment status or household size across our rural and urban respondents.

**Table 1:** Sample demographics and related characteristics.

Characteristic	Overall (n = 432)	Urban (n = 217)	Rural (n = 215)	P-value
Age (yrs.), mean (SD)	38.9 (14.6)	35.9 (12.1)	41.9 (16.2)	*<0.001
Gender, n (%)				*0.006
Male	144 (33.3)	59 (27.2)	85 (39.5)	
Female	288 (66.7)	158 (72.8)	130 (60.5)	
Education (highest level), n (%)				*<0.001
None	44 (10.2)	7 (3.2)	37 (17.2)	
Preschool	6 (1.4)	2 (0.9)	4 (1.9)	
Some primary	118 (27.3)	31 (14.3)	87 (40.1)	
Completed primary	61 (14.1)	25 (11.5)	36 (16.7)	
Some secondary	58 (13.4)	32 (14.8)	26 (12.1)	
O’ Level	57 (13.2)	42 (19.4)	15 (7.0)	
A’ Level	21 (4.9)	19 (8.8)	2 (0.9)	
Tertiary	45 (10.4)	37 (17.1)	8 (3.7)	
University degree	22 (5.1)	22 (10.1)	0 (0.0)	
Religion, n (%)				
Christian	350 (81.0)	167 (77.0)	183 (85.1)	*0.044
Muslim	82 (19.0)	50 (23.0)	32 (14.9)	
Ethnic group, n (%)				
Muganda	226 (52.3)	138 (63.6)	88 (40.9)	*<0.001
Munyankole	13 (3.0)	11 (5.1)	2 (0.9)	
Musoga	44 (10.2)	24 (11.1)	20 (9.3)	
Mukiga	5 (1.2)	2 (0.9)	3 (1.4)	
Iteso	37 (8.6)	8 (3.7)	29 (13.5)	
Bagisu	32 (7.4)	7 (3.2)	25 (11.6)	
Lugbara	2 (0.5)	2 (0.9)	0 (0.0)	
Other	73 (16.9)	25 (11.5)	48 (22.3)	
Marital status, n (%)				*<0.001
Married or living together	285 (66.0)	129 (59.5)	156 (72.6)	
Divorced/Separated	65 (15.1)	32 (14.8)	33 (15.4)	
Widowed	43 (10.0)	22 (10.1)	21 (9.8)	
Never married/lived together	39 (9.0)	34 (15.7)	5 (2.3)	
Employment status, n (%)				0.071
Employed	316 (73.2)	153 (70.5)	163 (75.8)	
Casual	6 (1.4)	1 (0.5)	5 (2.3)	
Unemployed	110 (25.3)	63 (29.0)	47 (21.9)	
Household size, mean (SD)	5.6 (3.5)	5.5 (3.3)	5.6 (3.7)	0.621
Wealth Quintile, n (%)				*<0.001
Lowest	13 (3.0)	0 (0.0)	13 (6.1)	
Low	28 (6.5)	0 (0.0)	28 (13.0)	
Middle	50 (11.6)	0 (0.0)	50 (23.3)	
High	63 (14.6)	4 (1.8)	59 (27.4)	
Highest	278 (64.4)	213 (98.2)	65 (30.2)	

SD = Standard deviation

* Significant at p < 0.05

Based on the relative best-minus-worst score method, we present the number of times each sector was selected as most or least important (best or worst), the mean best-worst scores, and standard errors in Table 2. Associated *t*-tests demonstrated that the mean best-worst scores for all sectors were different from zero, indicating that we were able to have adequate statistical power to measure respondents’ preferences for every sector. Overall, the health sector was the highest ranked sector (mean best-worst score = 0.793) where 82% of respondents selected health as the most important sector for the government to fund (n = 1417). The water and environment sector (0.495) and education sector (0.448) were ranked second (n = 1025, 59%) and third most (n = 903, 52%) important sectors for the government to invest in, respectively. This was followed by agriculture (0.398), works and transport (0.21), social development (0.186) and security (0.109). The two sectors selected as least important for government resource allocation were legislature (-0.508) and public administration (-0.477).

**Table 2:** Preferences for sector resource allocation from overall BWS survey sample.

Sector	Best (N)	Worst (N)	Mean Best-Worst score	SE	T-test†	P-value
Health	1417	45	0.793	0.011	71.051	<0.001
Water and Environment	1025	169	0.495	0.016	30.839	<0.001
Education	903	128	0.448	0.015	29.636	<0.001
Agriculture	903	218	0.396	0.016	23.496	<0.001
Works and Transport	608	244	0.210	0.016	13.068	<0.001
Social Development	649	326	0.186	0.017	10.677	<0.001
Security	502	312	0.109	0.016	6.744	<0.001
Energy and Mineral Development	159	388	-0.132	0.013	-10.071	<0.001
Public Sector Management	154	470	-0.182	0.013	-13.276	<0.001
Accountability	133	508	-0.217	0.013	-15.847	<0.001
Justice, Law, and Order	65	494	-0.248	0.012	-20.161	<0.001
Information and Communication Technology	166	608	-0.255	0.014	-17.187	<0.001
Lands, Housing, and Urban Development	68	589	-0.301	0.012	-23.295	<0.001
Tourism, Trade, and Industry	59	609	-0.318	0.012	-24.765	<0.001
Public Administration	46	871	-0.477	0.013	-36.060	<0.001
Legislature	55	933	-0.508	0.013	-37.706	<0.001

SE = Standard error

† t-test assessing whether each sector’s mean best-worst score is significantly different from zero

Comparing ranked preferences for resource allocation between urban and rural respondents, we observed similar results, where 1) health, 2) water and environment, and 3) education, and 4) agriculture sectors were the top four ranked sectors in both urban and rural areas (Table 3). Urban respondents ranked social development fifth (n = 341, 39%), while rural respondents preferred to invest in works and transport (n = 325, 38%) over social development (n = 308, 36%). Although the top ranked sectors were similar, two-sample t-tests show some differences between mean best-worst scores among urban and rural respondents. Health was more frequently chosen as important (n = 744, 86%) and less frequently chosen as least important (n = 17, 2%) among urban respondents. Energy and mineral development (rural -0.163; urban -0.101), and information and communication technology (rural -0.298; urban -0.213) sectors received significantly lower best-worst scores in rural areas where they were less preferred sectors for investment. Public administration (rural -0.410; urban -0.543), and legislature (rural -0.410; urban -0.604) were the least preferred for government investment in both rural and urban areas, but with significantly lower best-worst scores in urban areas.

**Table 3:** Preferences for sector resource allocation across urban and rural sub-samples.

Urban					Rural				
Sector	Best (N)	Worst (N)	Mean Best-Worst score	SE	Best (N)	Worst (N)	Mean Best-Worst score	SE	P-value†
Health	744	17	0.837	0.014	673	28	0.750	0.017	<0.001*
Water and Environment	515	84	0.496	0.022	510	85	0.494	0.022	0.950
Education	457	51	0.467	0.467	446	77	0.429	0.022	0.086
Agriculture	433	92	0.392	0.022	470	126	0.400	0.024	0.812
Social Development	341	164	0.203	0.024	308	162	0.169	0.023	0.331
Works and Transport	283	125	0.182	0.022	325	119	0.239	0.024	0.077
Security	249	164	0.097	0.023	253	148	0.122	0.022	0.443
Energy and Mineral Development	80	168	-0.101	0.017	79	220	-0.163	0.019	0.018*
Public Sector Management	87	244	-0.180	0.020	67	226	-0.184	0.018	0.884
Accountability	69	254	-0.213	0.019	64	254	-0.220	0.019	0.788
Information and Communication Technology	83	268	-0.213	0.020	83	340	-0.298	0.017	0.004*
Justice, Law, and Order	38	240	-0.232	0.017	27	254	-0.263	0.021	0.208
Lands, Housing, and Urban Development	34	285	-0.289	0.018	34	304	-0.313	0.018	0.354
Tourism, Trade, and Industry	32	292	-0.299	0.018	27	317	-0.337	0.018	0.138
Public Administration	13	485	-0.543	0.017	33	386	-0.410	0.019	<0.001*
Legislature	14	539	-0.604	0.017	41	394	-0.410	0.019	<0.001*

SE = Standard error

*Significant at p < 0.05

† Two sample t-test comparing mean BWS scores for urban and rural sub-samples

Table 4 summarizes our regression analysis results. In general, the regression analysis produced rankings of preferences for sectors similar to those obtained using the relative best-minus-worst score method analysis. However, there were some differences in preference order. For example, in the regression analysis, the education sector was ranked second followed by agriculture and water and development, whereas the relative best-minus-worst score method ranked water and development second, followed by education and agriculture. Health remained the most preferred sector to allocate resources to by a significant margin across both analyses.

**Table 4:** Conditional logistic regression estimates of preferences for sector resource allocation.

	Overall				Urban				Rural			
Sector	Est.	Rank	SE	P value	Est.	Rank	SE	P value	Est.	Rank	SE	P value
Health	3.027	1	0.064	<0.001*	3.333	1	0.099	<0.01*	2.822	1	0.083	<0.001*
Education	1.831	2	0.053	<0.001*	1.907	2	0.077	<0.01*	1.802	2	0.071	<0.001*
Agriculture	1.686	3	0.056	<0.001*	1.656	3	0.080	<0.01*	1.766	3	0.071	<0.001*
Water and Environment	1.611	4	0.054	<0.001*	1.643	4	0.078	<0.01*	1.605	4	0.070	<0.001*
Works and Transport	1.256	5	0.056	<0.001*	1.165	6	0.081	<0.01*	1.354	5	0.073	<0.001*
Social Development	1.100	6	0.054	<0.001*	1.183	5	0.078	<0.01*	1.057	6	0.072	<0.001*
Security	0.556	7	0.056	<0.001*	0.569	7	0.082	<0.01*	0.540	7	0.076	<0.001*
Energy and Mineral Development	-0.587	8	0.083	<0.001*	-0.523	8	0.116	<0.01*	-0.605	8	0.112	<0.001*
Information and Communication Technology	-0.731	9	0.084	<0.001*	-0.661	9	0.120	<0.01*	-0.724	9	0.114	<0.001*
Public Sector Management	-0.980	10	0.084	<0.001*	-0.815	10	0.114	<0.01*	-1.164	11	0.123	<0.001*
Accountability	-0.993	11	0.090	<0.001*	-0.911	11	0.127	<0.01*	-1.064	10	0.125	<0.001*
Justice, Law, and Order	-1.336	12	0.121	<0.001*	-1.164	12	0.160	<0.01*	-1.530	15	0.166	<0.001*
Lands, Housing, and Urban Development	-1.471	13	0.119	<0.001*	-1.412	13	0.169	<0.01*	-1.473	14	0.185	<0.001*
Tourism, Trade, and Industry	-1.600	14	0.127	<0.001*	-1.449	14	0.174	<0.01*	-1.720	16	0.185	<0.001*
Legislature	-1.655	15	0.131	<0.001*	-2.293	16	0.254	<0.01*	-1.308	12	0.153	<0.001*
Public Administration	-1.713	16	0.143	<0.001*	-2.228	15	0.265	<0.01*	-1.357	13	0.169	<0.001*

SE = Standard error

* Significant at p < 0.05.

**Fig 2:**
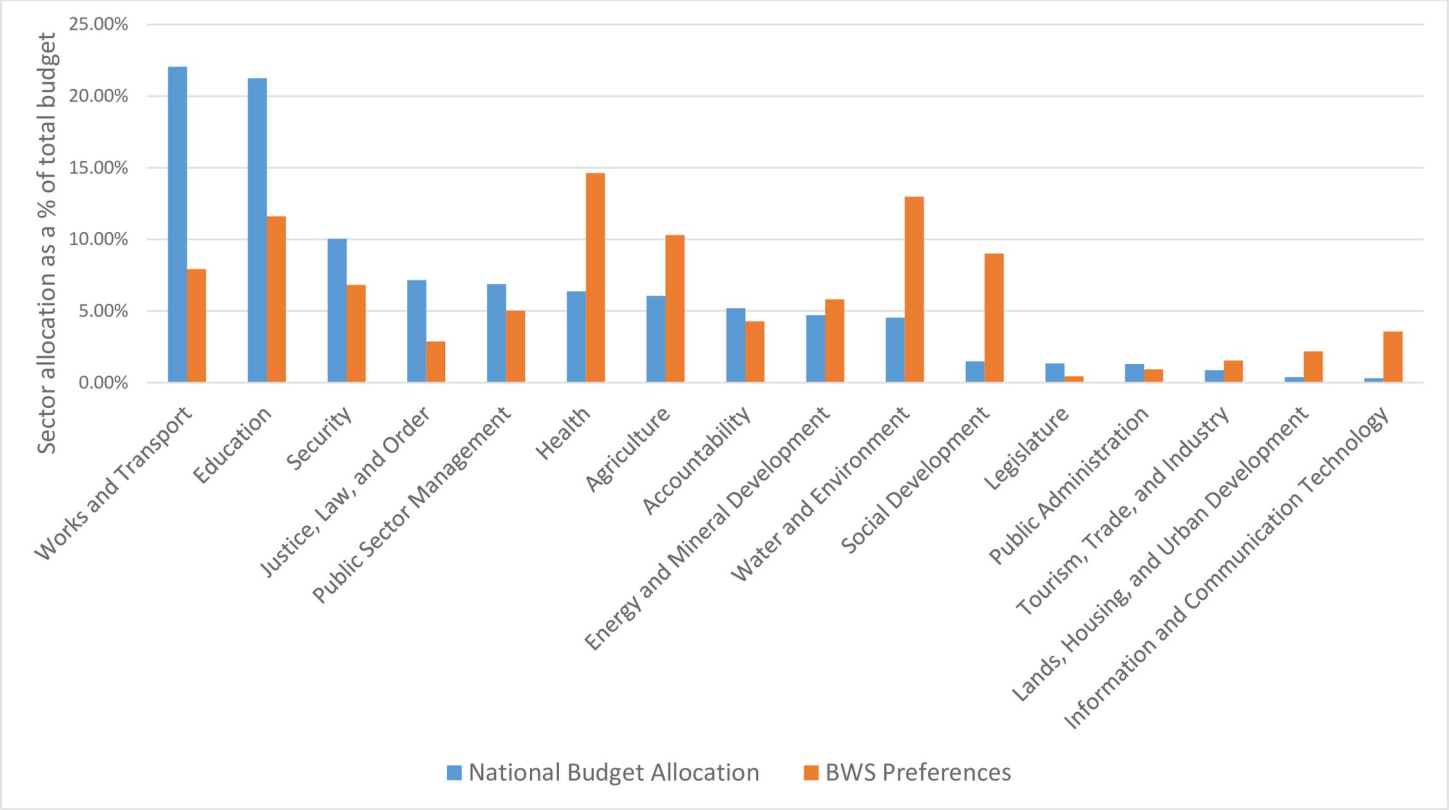
Overall BWS survey respondents' preferences for resource allocation and current national budget allocations across sectors.

We then compared respondent preferences for resource allocation from the relative best-minus-worst scores to the Uganda government’s national budget allocations for 2017–2018 [43] (Fig 2). While the health sector was ranked No. 1 for government resource allocations based on citizens’ preferences in the BWS survey, the health sector ranked sixth in actual national budget allocation, with only 6.4% of the total budget. The second ranked water and environment sector in the BWS survey ranked tenth with 4.6% of the national budget. Agriculture and social development similarly were ranked higher in the BWS survey (4^th^ and 6^th^, respectively) compared to the national budget allocation (7^th^– 6.1% and 11^th^– 1.5%, respectively). While information and communication technology was not ranked high in the BWS survey (12^th^), respondents still ranked it higher than its last (16^th^, 0.3%) ranking in the national budget.

Among sectors ranked in the top four in the BWS survey, only the education sector was in the top four ranking by national budget sector allocation. Overall, works and transport and education sectors appear to be receiving a disproportionately large proportion of the budget compared to respondents’ preferences (22.1% and 21.3% of the budget, respectively). Security, and justice, law and order also received large proportions of the budget (3^rd^ –10% and 4^th^– 7.2%, respectively) compared to citizens’ rankings (7^th^ and 12^th^, respectively).

## Discussion

Our finding that more than 80% of surveyed respondents ranked the health sector as the most important sector for the government to allocate resources in demonstrates the importance of making investments in the health sector even among other competing demands. This result should empower policy makers and stakeholders to better advocate for more financing for health, not only to meet the government’s health commitments and goals, but also to satisfy citizens’ needs and preferences.

Despite people’s strong preferences to invest in health, the health sector remains significantly underfunded. The proportion of the Uganda national budget spent on health is at 6.4%, which is less than half of the 15% target agreed upon in the Abuja declaration [44, 45]. Moreover, this figure already includes external financing, which masks the Ugandan government’s domestic resource allocations and spending. In fact, almost half (909.62 billion Ugandan shillings, US$242 million) of the total health sector allocation (1,824.08 billion Ugandan shillings, US$487 million) is from external financing, which suggests that domestic resources for health constitute only 3.2% of the national budget [43]. In the context of declining and uncertain foreign funding flows [5], it is imperative that Ugandan policy makers mobilize more domestic resources and create more fiscal space for the health sector.

Our study found that respondents’ preferences for resource allocation across sectors were generally misaligned with current government budgetary allocations. Beyond health, water and environment, agriculture, and social development sectors were largely underfunded compared to respondents’ preferences. Works and transport, education, security, and justice, law and order received a larger share of the national budget compared to people’s preferences. Reallocation of resources to meet citizens’ preferences would not only improve social services in the country, but also better respond to citizens’ choices and needs.

Strong preferences among respondents for resource allocation to social sectors should encourage policy makers and other stakeholders to take a holistic approach to invest across social sectors. Education, water and environment sectors, for example, have synergistic relationships with the health sector [46–48], contributing to health system strengthening and improving health outcomes through the life course [49]. Moreover, findings on the heterogeneous nature of preferences for resource allocation among rural and urban county respondents underscore the need for a tailored approach to resource planning that is responsive to local community contexts and preferences.

Our study findings are comparable to the few published studies eliciting citizens’ preferences for resource allocation in the African region [28]. One study in Kenya elicited citizens’ preferences for sector level spending by asking respondents directly what proportion of the budget they believed should be allocated to each sector and compared the mean proportions to actual expenditures [50]. Similar to our results, the study found that social sectors, including the health sector, were underfunded in the Kenya national budget relative to study respondents’ preferences. In Uganda, a study elicited citizen preferences for foreign aid programs compared to government programs for development projects, and found that citizens were more willing to support foreign aid projects compared to government programs [51].

We note several limitations to our study and its findings. First, our study utilized a cross-sectional design and participants were drawn from one district (Mukono) in Uganda. Thus our findings may not be representative of the general Uganda population or reflect changes in preferences over time. Further studies with nationally representative samples as well as longitudinal studies may explore multisectoral resource allocation preference at the national level, and explore any changes in preferences over time. Second, while the BWS survey method has several advantages and is easier to understand compared to other preference elicitation techniques [52], it may still be difficult for respondents to interpret the choice tasks, especially for rural populations and respondents with low education levels. To mitigate this impact, we employed visual aids illustrating choice tasks and incorporated a simple non-study related warm-up choice task based on food preferences to aid respondents’ understanding. While we pre-tested the visual aids for comprehension, use of a single picture to represent sectors, whose work is complex, could have inadvertently introduced some bias emanating from individuals’ perceptions of representative pictures.

In addition, while national budget plans and planned sector allocations are strong indicators of governments’ policy preferences, they are still imperfect measures of actual spending and are subject to the government meeting its domestic revenue and external financing targets. Thus, the ranking of the sectors in resource allocation presented in our analysis may not reflect the actual expenditures or supplementary budgetary processes that occurred within the budget year. Furthermore, citizen preferences are not the sole criterion in government resource allocation decision making. While evidence of citizen preferences can be a strong advocacy tool, a multitude of other socio-economic and political factors influence resource allocations. Finally, our study occurred during a period of contentious debate and political acrimony over a proposed law to remove constitutional clauses on presidential term limits [53]. It is possible that the political atmosphere at the time of the study and in-person data collection through research assistants could have led to reticence among respondents in expressing their preferences or heightened social desirability bias. Despite these limitations, we believe this study makes a unique and valuable contribution by eliciting citizens’ preferences to inform health policy.

## Conclusion

This study is among the first to elicit preferences for resources allocation across sectors using the BWS method and compare this with government budgetary allocations. The study adds to the limited number of BWS studies in sub-Saharan Africa to elicit preferences to inform policy [28]. By demonstrating how health ranks against other sectors, our study results call for greater investment in health in Uganda to meet citizens’ preferences. The results also support greater collaboration in agenda setting and resource planning between fiscal and health authorities in African countries and other LMICs [54, 55]. In the context of multilateral goals in the Abuja declaration and recommendations of the High Level Task Force on Innovative International Financing for Health Systems [56, 57], evidence of strong preferences by citizens to allocate resources to the health sector could help make the case for increased health sector allocations. As external funding for health declines globally and Ugandan citizens’ healthcare access and needs grow with development, it is vital for the Ugandan government to mobilize more domestic resources for health.

## Supporting information

S1 File Study survey questionnaire. (PDF)

S1 Data Study dataset. (DTA)

S1 Fig (XLSX)
