# Supplementary material for: Health versus other sectors: Multisectoral resource allocation preferences in Mukono district, Uganda
Source: PLoS One. 2020 Jul 30;15(7):e0235250. doi: 10.1371/journal.pone.0235250 (PMC7392331; doi:10.1371/journal.pone.0235250)
Supplement: S1 File — (PDF) [file pone.0235250.s001.pdf]

# Best Worst Scaling

Study ID ________________________________

**Let us do an example with food choices. You have a limited amount of money to buy food and cannot buy everything. There are four choices: Ugali, Matoke, Chapati, and Meat. Which food is most important in your diet? Which food is least important in your diet?**

| | Ugali | Matoke | Chapati | Meat |
|---|---|---|---|---|
| Most Important | ○ | ○ | ○ | ○ |
| Least Important | ○ | ○ | ○ | ○ |

**Now I am going to show you groups of some of the sectors which we discussed. I will show you different groups many times. Remember there is not enough money to spend on all programs in every sector and we have to prioritize what sectors and programs to invest in. Each time I show you a group of sectors, please select one sector that would be the most important for you and select one sector that would be the least important to you and your community, when deciding what to invest limited resources in.**

| | Works and Transport | Justice, Law, and Order | Public Sector Management | Public administration |
|---|---|---|---|---|
| Most Important | ○ | ○ | ○ | ○ |
| Least Important | ○ | ○ | ○ | ○ |

| | Education | Health | Accountability | Public Administration |
|---|---|---|---|---|
| Most Important | ○ | ○ | ○ | ○ |
| Least Important | ○ | ○ | ○ | ○ |

| | Water and Environment | Tourism, Trade, and Industry | Lands, Housing, and Urban Development | Public Administration |
|---|---|---|---|---|
| Most Important | ○ | ○ | ○ | ○ |
| Least Important | ○ | ○ | ○ | ○ |

| | Security | Water and Environment | Justice, Law, and Order | Legislature |
|---|---|---|---|---|
| Most Important | ○ | ○ | ○ | ○ |
| Least Important | ○ | ○ | ○ | ○ |

| | Works and Transport | Energy and Mineral Development | Tourism, Trade, and Industry | Social Development |
|---|---|---|---|---|
| Most Important | ○ | ○ | ○ | ○ |
| Least Important | ○ | ○ | ○ | ○ |

| | Security | Works and Transport | Health | Information and Communication Technology |
|---|---|---|---|---|
| Most Important | ○ | ○ | ○ | ○ |
| Least Important | ○ | ○ | ○ | ○ |

| | Education | Energy and Mineral Development | Public Sector Management | Legislature |
|---|---|---|---|---|
| Most Important | ○ | ○ | ○ | ○ |
| Least Important | ○ | ○ | ○ | ○ |

| | Security | Accountability | Tourism, Trade, and Industry | Public Sector Management |
|---|---|---|---|---|
| Most Important | ○ | ○ | ○ | ○ |
| Least Important | ○ | ○ | ○ | ○ |

| | Water and Environment | Accountability | Energy and Mineral Development | Information and Communication Technology |
|---|---|---|---|---|
| Most Important | ○ | ○ | ○ | ○ |

| | | | | |
|---|---|---|---|---|
| Least Important | ○ | ○ | ○ | ○ |

| | Agriculture | Justice, Law, and Order | Accountability | Social Development |
|---|---|---|---|---|
| Most Important | ○ | ○ | ○ | ○ |
| Least Important | ○ | ○ | ○ | ○ |

| | Works and Transport | Agriculture | Education | Water and Environment |
|---|---|---|---|---|
| Most Important | ○ | ○ | ○ | ○ |
| Least Important | ○ | ○ | ○ | ○ |

| | Health | Justice, Law, and Order | Energy and Mineral Development | Lands, Housing, and Urban Development |
|---|---|---|---|---|
| Most Important | ○ | ○ | ○ | ○ |
| Least Important | ○ | ○ | ○ | ○ |

| | Agriculture | Lands, Housing, and Urban Development | Information and Communication Technology | Public Sector Management |
|---|---|---|---|---|
| Most Important | ○ | ○ | ○ | ○ |
| Least Important | ○ | ○ | ○ | ○ |

| | Social Development | Information and Communication Technology | Public Administration | Legislature |
|---|---|---|---|---|
| Most Important | ○ | ○ | ○ | ○ |
| Least Important | ○ | ○ | ○ | ○ |

| | Agriculture | Health | Tourism, Trade, and Industry | Legislature |
|---|---|---|---|---|
| Most Important | ◯ | ◯ | ◯ | ◯ |
| Least Important | ◯ | ◯ | ◯ | ◯ |

| | Security | Education | Lands, Housing, and Urban Development | Social Development |
|---|---|---|---|---|
| Most Important | ◯ | ◯ | ◯ | ◯ |
| Least Important | ◯ | ◯ | ◯ | ◯ |
